# Vitamin K1 Administration Increases the Level of Circulating Carboxylated Osteocalcin in Critically Ill Patients

**DOI:** 10.3390/nu17020348

**Published:** 2025-01-19

**Authors:** Nadide Aydin, Thomas Kander, Ulf Schött, Sassan Hafizi

**Affiliations:** 1 School of Medicine, Pharmacy and Biomedical Sciences, University of Portsmouth, Portsmouth PO1 2DT, UK; 2 Department of Clinical Sciences, Lund, Institution for Anaesthesiology & Intensive Care, Lund University, 22185 Lund, Sweden; thomas.kander@med.lu.se; 3 Department of Intensive and Perioperative Care, Skåne University Hospital, 22185 Lund, Sweden

**Keywords:** vitamin K, osteocalcin, Gas6, PIVKA-II, carboxylation, intensive care, critical illness, inflammation, sepsis

## Abstract

Background/Objectives: Vitamin K-dependent proteins (VKDPs) all commonly possess specially modified γ-carboxyglutamic acid residues created in a vitamin K-dependent manner. Several liver-derived coagulation factors are well characterised VKDPs. However, much less is known about extrahepatic VKDPs, which are more diverse in their molecular structures and functions, and some of which have been implicated in inflammatory disorders. Vitamin K metabolism was shown to be impaired in critically ill patients, in whom systemic inflammation and sepsis are common features. Therefore, the aim of this study was to investigate the effect of vitamin K administration to these patients on their circulating levels of selected VKDPs. A particular novelty of this study was the measurement of specifically carboxylated forms of these proteins in addition to their overall levels. Methods: Blood samples were taken from 47 patients in the intensive care unit before and approximately 24 h after intravenous vitamin K1 (10 mg) administration, and proteins were analysed by specific immunoassay. Results: Vitamin K1 induced increases in plasma levels of carboxylated osteocalcin and total Gas6 (*p* = 0.0002 and *p* = 0.0032, respectively). No changes were detected in levels of carboxylated Gas6 or PIVKA-II (undercarboxylated prothrombin), although the latter positively correlated with undercarboxylated osteocalcin (r = 0.38). Conclusion: Injected vitamin K1 increases the blood levels of two distinct VKDPs in critically ill patients, both of which have been implicated in inflammation regulation, including the increased carboxylation of one of them.

## 1. Introduction

Vitamin K, a member of the fat-soluble vitamins, plays a crucial role in various physiological processes, including blood coagulation, bone metabolism, tissue calcification and antioxidant activity [1]. A key biochemical role of vitamin K is as a cofactor to the enzyme gamma-glutamyl carboxylase (GGCX), which modifies glutamic acid (Glu) residues to γ-carboxyglutamic acid (Gla) within so-called vitamin K-dependent proteins (VKDPs) in the endoplasmic reticulum [2]. The human body is not able to store a high amount of vitamin K, which is rapidly depleted. However, vitamin K can be reused many times through a two-step process in the vitamin K cycle. Concomitant with the carboxylation of Glu residues, GGCX activity oxidises vitamin K hydroquinone (KH_2_) to vitamin K 2,3-epoxide (KO). Then, the enzymes vitamin K epoxide reductase (VKORC1; canonical vitamin K cycle) and ferroptosis suppressor protein 1 (FSP1; non-canonical vitamin K cycle) reduce KO to KH_2_ in reactions that are, respectively, sensitive or insensitive to warfarin inhibition [3,4].

The γ-carboxylation by GGCX is a major post-translational modification in VKDPs that confers the ability to coordinate calcium ions, which is key to their functions. VKDPs can be categorised into two groups, hepatic and extrahepatic. Hepatic VKDPs include the coagulation factors: factor II (prothrombin), VII, IX, X and proteins C, S, Z. These proteins are homologous and their vitamin K-modified, calcium-bound forms are able to associate with negatively charged membrane phospholipids such as are present on activated cell surfaces during injury, including platelets, endothelial cells and apoptotic cells [5]. Extrahepatic VKDPs include proteins that are not homologous such as Gas6, osteocalcin (OC), matrix Gla protein (MGP), Gla-rich protein (GRP), periostin and periostin-like-factor, as well as four other Gla-containing proteins whose functions are currently unknown [6]. This group of VKDPs is more diverse in their compositions; however, they utilise their common high affinity for calcium to perform distinct functions in a variety of physiological processes in extrahepatic tissues, including bone metabolism, vascular repair and cell survival [6].

The process of carboxylation may also represent a difference in vitamin K metabolism between the liver and extrahepatic tissues. For example, the activity of the vitamin K recycling enzyme VKORC1 is three times higher in vascular smooth muscle cells than in the liver, suggesting a greater reliance on vitamin K reserves for VKDP carboxylation in the periphery than in the liver [7]. Furthermore, we have recently shown in rodent brain tissues and cells the presence of a functional vitamin K cycle that carboxylates Gas6 [8]. In addition, the menaquinone forms of vitamin K (K2) have been suggested to be more important for peripheral carboxylation than phylloquinone (K1), which the liver primarily utilises [9]. However, this distinction is not certain as K1 and K2 have both been shown to reduce the level of undercarboxylated OC in postmenopausal women [10]. However, there is a need for a better understanding of the extrahepatic role of vitamin K through its effects on the functionality of this lesser-known grouping of VKDPs.

Despite the recycling of vitamin K within tissues, deficiency or blockade of vitamin K function can occur in certain situations, such as in hospitalised patients. This would be expected to lead to uncarboxylation or undercarboxylation of VKDPs, also referred to as ‘proteins induced in vitamin K absence or antagonism’ (PIVKA), which would result in reduced biological activity. For instance, in patients administered warfarin, which targets VKORC1 and is the most commonly used vitamin K antagonist [2], there may be increased levels of PIVKAs in addition to the clotting factors. Other factors that could result in vitamin K deficiency in hospitalised patients include insufficient supply (pre- and intra-hospital malnutrition, prolonged intravenous nutrition), malabsorption (bowel inflammation, gastric retention, sepsis or ischaemic induced intestinal wall damage), drugs, increased vitamin K usage seen during acute illness and genetic factors [9]. Moreover, under these conditions, elevated PIVKA levels may be increased without coagulation being affected, and this can often be interpreted as subclinical vitamin K deficiency. Therefore, greater knowledge of the carboxylation status of extrahepatic VKDPs may provide greater clarity on the true overall clinical status of patients even with normal coagulation.

In critically ill patients, the status of vitamin K metabolism was shown to be profoundly impaired, affecting >20% of intensive care unit (ICU) patients [11]. In an excellent recent review by Paulus et al. [11], several illustrations show the pathophysiology behind vitamin K deficiencies in critically ill patients. Furthermore, there is some evidence that vitamin K deficiency may correlate with prolonged ICU stays, mechanical ventilation, and increased mortality. Our group has previously reported that ICU patients exhibit higher blood levels of PIVKA-II, also known as des-γ-carboxy-prothrombin, upon admission than healthy adults [12]. Gas6 is a widely expressed VKDP that is a ligand for TAM receptor tyrosine kinases [13], through which it activates signalling for cell survival as well as suppression/resolution of inflammatory and immune responses [14]. Elevated levels of Gas6 were observed in patients with severe sepsis [15]. We reported that intravenous vitamin K1 administration to patients in intensive care resulted in an increase in plasma concentrations of Gas6 protein after 24 h [16]. Osteocalcin (OC) is an abundant bone VKDP produced in osteoblasts, with three Gla residues that bind to calcium ions in hydroxyapatite. However, more recent studies also implicate broader roles for OC in metabolism, reproduction and cognition [17]. Circulating OC levels were found to be reduced in patients with severe COVID-19 compared with non-COVID-19 controls [18]. However, the carboxylation status of OC in critically ill patients has not been determined. Therefore, knowledge of circulating levels of VKDPs alongside vitamin K deficiency may present a new avenue for the management of critical illness. However, the measurement of absolute levels of VKDPs does not address the principal biochemical role of vitamin K as a cofactor to GGCX. Therefore, greater clarity and more valuable information may come from establishing the carboxylation status of these VKDPs alongside their absolute levels.

No study to date has investigated the post-translational modification status, through γ-carboxylation, of Gas6 and osteocalcin in critically ill patients, nor the impact of vitamin K administration on these. In the present study, we addressed these gaps, using the same cohort of ICU patients from our previous study who were administered intravenous vitamin K1 [16]. Furthermore, this is the first study of Gas6 carboxylation status in any clinical scenario. Such new insights are needed to further elucidate the mechanisms behind the impact of subclinical vitamin K deficiency on critically ill patients and, therefore, strengthen the rationale for using vitamin K as a therapy.

## 2. Materials and Methods

### 2.1. Study Design

The samples for this study came from a previously published main study [19], which took place at the general ICU of Skåne University Hospital, Lund, Sweden. Ethical approval for the study was provided by the Regional Ethics Board (20 December 2018; DNR 2018/1010). Written informed consent was obtained from all patients or closest relative. The study is registered at ClinicalTrials.gov (identifier NTC3782025). Both the original study [19] and the first substudy of Gas6/Axl [16] included 52 patients; a sample size of 50 had a 90% power with sample size calculation using G*Power version 3.1 (Heinrich Heine Universität, Düsseldorf, Germany) [19].

All patients were treated according to standard procedures at the ICU. The inclusion criteria for study participation were patients aged 18 or older with elevated routine Owren PT-international normalised ratio (PT-INR > 1.2) and prescribed 10 mg intravenous vitamin K1 (Konakion^®^) by their ICU physician. The exclusion criteria were patients prescribed warfarin or novel oral anticoagulants, or who had been prescribed prothrombin complex concentrate and plasma, or who had already been treated with Konakion® in the previous 36 h. The study also excluded patients with known hereditary coagulative disorders and those with hepatocellular carcinoma or liver resection within the previous 6 months. Patients were also categorised according to sequential organ failure assessment (SOFA) scores [20]. Samples were taken both prior to as well as 20–28 h after vitamin K1 administration, a period which we have previously reported to improve PT-INR in ICU patients not on warfarin [20].

### 2.2. Enzyme-Linked Immunosorbent Assays (ELISAs) for the Quantification of VKDPs in Human Plasma

Citrate plasma samples were analysed by enzyme-linked immunosorbent assays (ELISA) according to the assay manufacturer’s instructions for the following proteins: total Gas6 (R&D Systems, Bio-Techne, Minneapolis, MN, USA), γ-carboxylated Gas6 (Gla-Gas6), undercarboxylated osteocalcin (ucOC; Novus Biologicals, Bio-Techne), carboxylated osteocalcin (Gla-OC; Takara Bio, London, UK), and PIVKA-II (Stratech, Ely, UK).

The Gla-Gas6 ELISA was developed in-house for the detection of specifically γ-carboxylated (Gla) residues in Gas6 and has previously been described for analysis of mouse Gas6 protein [8]. In the present study, in order to determine the comparative degrees of carboxylation of Gas6 across patient samples, the samples were first assayed for total Gas6. The results from this analysis then enabled appropriate dilution of each sample so as to make the total Gas6 concentrations equal across all samples. Subsequently, samples were assayed for Gla-Gas6. In the human Gla-Gas6 ELISA, a 96-well microplate with high protein binding capacity (NuncTM Maxisorp, Thermo Fisher Scientific, Loughborough, UK) was first coated with anti-human Gas6 antibody (1 µg/mL, R&D Systems) diluted in a coating buffer (30 mM Na_2_CO_3_, 200 mM NaHCO_3_, pH 9.0), and the plate was incubated overnight at 4 °C. For all ELISAs, all plate washing steps included three repeat washes with 400 µL per well of wash buffer. Plates were blocked with 2% BSA for 2 h at RT. After a washing step, plasma samples were added to the plates and incubated overnight at 4 °C. Samples were diluted in PBS + 0.1% BSA at 1:100 for total Gas6, Gla-Gas6 and ucOC assays and 1:20 for Gla-OC and PIVKA-II assays. After incubation, samples were removed from wells and the plates were washed, and the detection antibody was added. For the Gla-Gas6 assay, this was mouse anti-Gla (mAb 3570; Biomedica Diagnostics, Stamford, CT, USA), diluted (0.25 μg/mL) in antibody dilution buffer (PBS, 0.1%, BSA%, 0.05% Tween-20) and incubated for a minimum of 3 h at RT. The solution was removed and anti-mouse HRP antibody in antibody dilution buffer (1:2000) was added to each well and incubated for minimum 1.5 h at RT.

In the final stage for all ELISAs, the plates were washed and substrate solution was added, which was a 1:1 mix of Colour Reagent A (H_2_O_2_) and Colour Reagent B (3,3′,5,5′-tetramethylbenzidine; TMB) (Pierce TMB Substrate Kit, Thermo Fisher Scientific). The plate was protected from direct light during colour development; a stop solution (2 M H_2_SO_4_) was finally added to the wells, and optical density (OD) was measured with a microplate reader (SpectraMax i3x, Molecular Devices, San Jose, CA, USA) at wavelengths 450 nm and 570 nm. In all ELISA assays, the measured values were blanked to eliminate background interference by subtracting the 450 nm OD value from the 570 nm value; the OD value of the zero standard was subsequently also subtracted.

### 2.3. Statistical Analyses

Statistical analysis and graphs were performed using Prism software v.10.4.1 (GraphPad Software Inc., San Diego, CA, USA) to determine significance for paired comparisons using Wilcoxon matched-pairs (non-parametric) or paired *t*-test (parametric). For correlation analysis, the coefficient of determination (r) and statistical significance (*p*-value) were determined using Pearson’s coefficient for variables that were normally distributed and continuous, and Spearman correlation for those that were not. All data are shown with statistical significance based on the following criteria: * *p* < 0.05 and *** *p* < 0.001 unless otherwise stated in the accompanying figure legends.

## 3. Results

### 3.1. Patient Population

The study analysed blood samples from ICU patients (frozen plasma Eppendorf cuvettes), with n = 47 ‘before’ and ‘after’ pairs (total 94 samples) unless otherwise stated for a particular assay. The fewer samples analysed in those cases compared to the previous study [19] was due to a lack of sufficient sample amount for a complete sample pair for certain patients. The participant male:female ratio was 67%:33%, and the median age was 67 years (range: 20–86 years). The most common diagnosis was septic shock (29%) followed by cardiovascular disease (13%). Three patients (6%) were diagnosed with cancer.

### 3.2. Total Gas6 and Gla-Gas6 Plasma Concentrations Before and After Vitamin K

**Figure 1:**
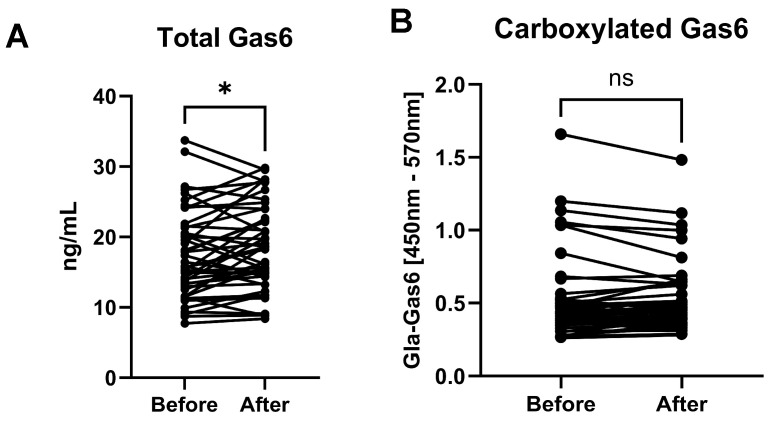
Detection of total Gas6 (**A**) and γ-carboxylated Gas6 (Gla-Gas6; (**B**)) in blood plasma of ICU patients before and after vitamin K injection. Gla-Gas6 was quantified in samples all as appropriately diluted to contain equal concentrations of total Gas6. Total Gas6 is significantly increased by vitamin K. Statistical significance was determined using paired *t*-test; * p = 0.0318, ns = not significant, for comparisons indicated by lines (n = 43).

Total Gas6 in patient samples was increased after vitamin K injection (Figure 1A). Next, equal total Gas6 concentrations across all samples were assayed for Gla-Gas6. The results showed no difference in Gla-Gas6 signal between patient sample pairs (i.e., before vs. after vitamin K injection) (Figure 1B).

### 3.3. Undercarboxylated and Carboxylated Osteocalcin Plasma Concentrations Before and After Vitamin K

**Figure 2:**
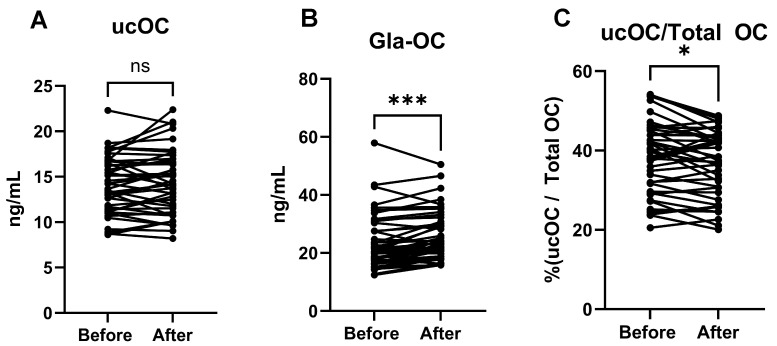
Detection of undercarboxylated osteocalcin (ucOC; (**A**)) and carboxylated osteocalcin (Gla-OC; (**B**)) in blood plasma of ICU patients before and after vitamin K injection. Gla-OC is significantly increased after vitamin K injection. Also, the proportion of ucOC within total OC in samples is significantly decreased after vitamin K injection (**C**). Statistical analysis was determined using paired *t*-test for ucOC parametric data (n = 43 pairs) and Wilcoxon match-pair test for Gla-OC non-parametric data (n = 47 pairs) for comparisons indicated by lines; * *p* < 0.05, *** *p* < 0.001, ns = not significant.

The undercarboxylated osteocalcin assay (ucOC) showed no difference within sample pairs (n = 43) (Figure 2A). In contrast, the carboxylated osteocalcin assay (Gla-OC) showed a clear elevation of Gla-OC in post-vitamin K samples (*p* = 0.0002) (Figure 2B). Furthermore, the proportion of total OC that was non-carboxylated was reduced in post-vitamin K samples (*p* = 0.014) (Figure 2C).

### 3.4. PIVKA-II Plasma Concentrations Before and After Vitamin K

**Figure 3:**
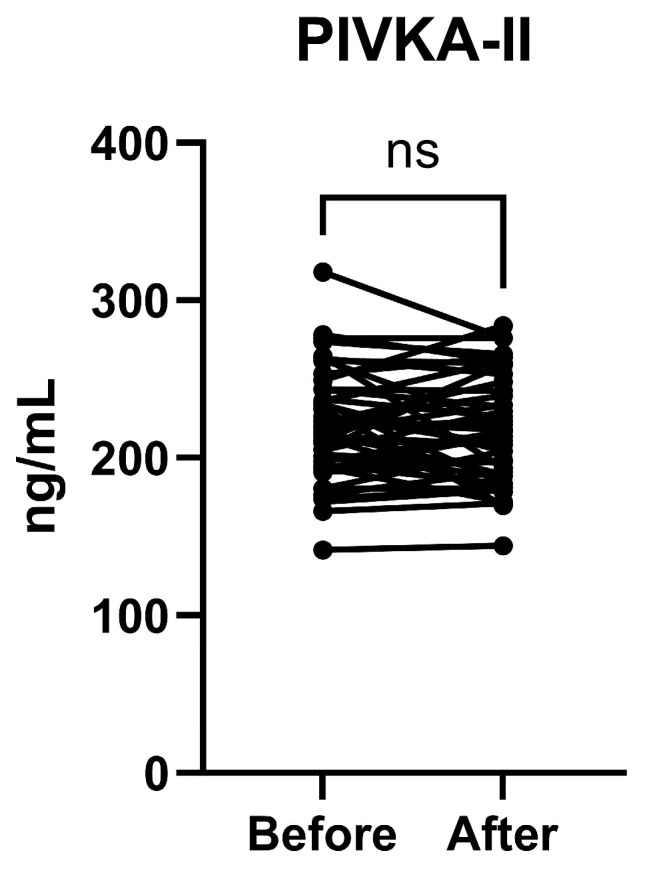
Detection of PIVKA-II in blood plasma of ICU patients before and after vitamin K injection. Statistical analysis was determined using paired *t*-test) for comparisons indicated by lines; ns = not significant (n = 47 pairs).

No difference was measured between the ‘before’ and ‘after’ sample pairs in the plasma concentration of PIVKA-II (Figure 3).

In addition, the data were analysed extracting those for three distinct disease categories common amongst patients in ICU: septic shock, which formed the largest proportion (n = 14), cardiovascular disease (n = 6), and cancer (n = 2) (sample numbers were those that had a complete sample pairing measured). The remaining patients had other main diagnoses. Despite the expectedly lower sample numbers per subgroup, the analysis nevertheless showed that in the septic shock group, there was still a statistically significant difference between the ‘Before’ and ‘After’ groups for Gla-OC (*p* = 0.0353). Therefore, this indicates that the vitamin K1 effects observed for the entire sample set apply specifically to at least the subset of patients with septic shock.

### 3.5. Correlations Between Analyte Levels in This Study

**Figure 4:**
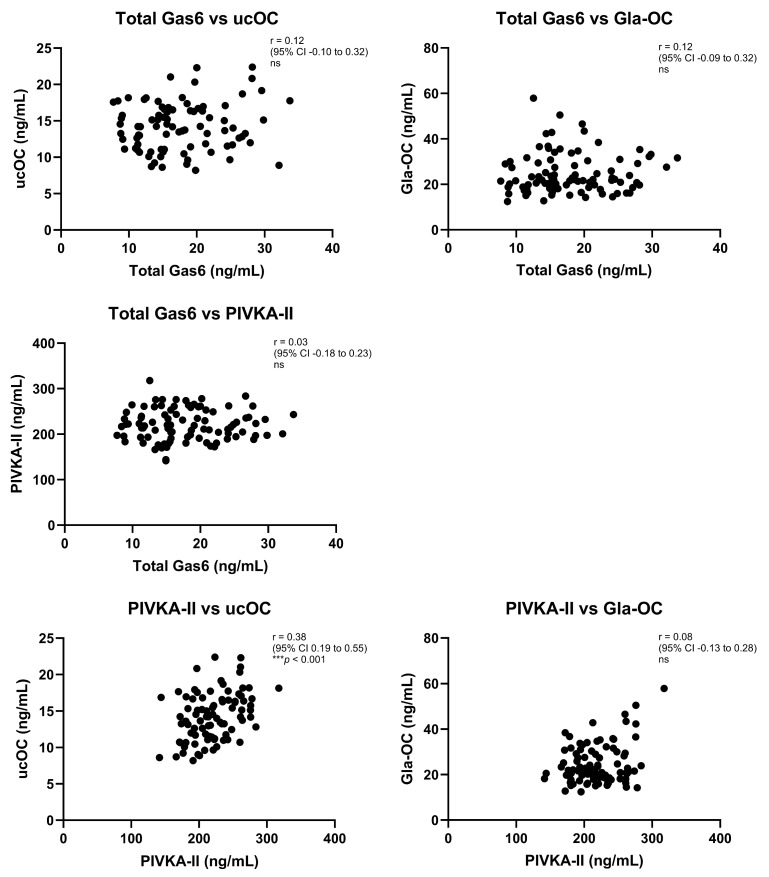
Correlation analyses were performed between total Gas6, undercarboxylated osteocalcin (ucOC), carboxylated osteocalcin (Gla-OC) and PIVKA-II. The coefficient of determination (r) and statistical significance (*p*-value) were determined by Pearson correlation analysis, except for Gla-OC data, which were not normally distributed and hence Spearman correlation was applied. N = number of XY pairs representing both ‘before’ and ‘after’ samples from patients. Total Gas6 vs. ucOC (n = 86); total Gas6 vs. Gla-OC (n = 94); total Gas6 vs. PIVKA-II (n = 94); PIVKA-II vs. ucOC (n = 86); PIVKA-II vs. Gla-OC (n = 94). *** *p* < 0.001, ns = not significant.

Correlation analysis amongst all analytes revealed a positive correlation between PIVKA-II and ucOC levels (Figure 4). No correlations were found between any other analytes.

## 4. Discussion

Subclinical vitamin K deficit is common in critically ill patients and in the perioperative period. The causes for this could be multifactorial, including insufficient dietary supply or impairments in intestinal uptake or transport from the liver [11]. Vitamin K deficiency is traditionally defined as prothrombin Owren values within the normal range of 0.9–1.2 and an increased PIVKA plasma level [12]. However, more reliable information on the vitamin K status of patients could be provided by tests that are more targeted, for example, analysing the levels of carboxylated forms of VKDPs. We have previously observed in patients undergoing major surgery that circulating levels of desphospho-uncarboxylated-MGP (dp-ucMGP) were higher than reference values preoperatively, with the levels increasing further four days after the surgery [21]. We then investigated ICU patients administered vitamin K1 through intravenous injection and detected a decrease in dp-ucMGP blood levels after 24 h [19], indicating that exogenous vitamin K1 induces an increase in VKDP carboxylation in patients. In another study, cardiovascular risk patients had higher dp-ucMGP preoperatively [21], possibly indicating an unhealthy diet with low vitamin K intake (no vegetables or leafy greens) [22]. We subsequently reported that vitamin K1 caused an increase in plasma concentrations of total Gas6 protein, although the Gas6 carboxylation state was not known [16]. In the present study, we investigated the carboxylated forms of three distinct VKDPs in the circulation of patients in the ICU, both before and after intravenous administration of vitamin K1. Furthermore, this is the first report of the detection of carboxylated Gas6 (Gla-Gas6) in clinical samples.

Our results showed that vitamin K1 injection increased total Gas6 protein levels. This is concomitant with increased soluble Axl (Gas6 receptor) in the same cohort [16]. Blood levels of both proteins have previously been shown to positively correlate with inflammatory markers in patients with sepsis [15]. These observations suggest that activation of the Gas6/Axl system occurs as a pro-resolution response to systemic inflammation, as this system is recognised as an anti-inflammatory signalling pathway [13,14]. Moreover, the post-vitamin K injection Gas6 levels were increased both in patients with increased CRP and those with decreased SOFA score (i.e., better clinical outcomes) [19]. Thus, these observations suggest a clinically induced anti-inflammatory response. The transcriptional regulatory mechanisms of vitamin K1 action are not well known. However, MK-4 has been identified to bind to the human SXR nuclear receptor and upregulate MGP [23]. Therefore, K1 may either regulate transcription directly or via conversion to K2, and this may also apply to the expression of other VKDPs, although this remains to be studied.

While Gas6 levels increased after vitamin K1 injection, we observed no significant change in levels of Gla-Gas6, which indicates that the degree of carboxylation per Gas6 molecule did not change. It is possible that all Gas6 in the blood of these patients was already fully carboxylated (i.e., all 11 Gla residues), such that added vitamin K would have no further effect. However, our detection of undercarboxylated OC (ucOC) and PIVKA-II in samples revealed that incomplete VKDP carboxylation is present in those patients’ plasma. Another possibility is that our Gla-Gas6 assay could not with sufficient precision detect differences in the degree of Gas6 carboxylation between sample pairs that may have differed by, e.g., one or two Gla residues. Alternatively, differences may also not have been detected between Gas6 molecules that had the same total numbers of Gla residues but which were distributed differently across the total eleven residues, and which could have functional consequences. These unknowns represent a technical limitation of our ELISA, which utilised a pan-Gla antibody that recognises the modified amino acid in multiple VKDPs and in varied sequence contexts although without further precision. Mass spectrometry is able to determine with precision the sequence placement of carboxylation that is present in proteins; however, this methodology remains to be optimised for analysing VKDPs in blood. Nevertheless, the observation of an increase in circulating Gas6 protein induced by vitamin K1 injection shows a clear physiological response. This may have occurred through activation of the vitamin K cycle machinery or instead through other more direct transcriptional effects of vitamin K1.

The present study also revealed a clear increase in carboxylated osteocalcin (Gla-OC) in patients following vitamin K1 injection. Furthermore, subgroup analysis of the data revealed that the pro-carboxylation effect of injected vitamin K1 occurs at least amongst the patients with septic shock (Supplementary Table S1. Assay results on patient subgroups according to disease category). In previous human studies, circulating OC levels were found to be decreased during critical illness, which correlated with increased bone resorption biomarkers [24], whilst they were increased in the recovery period [24,25]. In critically ill patients with COVID-19, circulating OC levels were reduced compared with non-COVID-19 controls, as well as associated with longer ICU stays [18,26]. Extending to other vitamin K markers, extrahepatic vitamin K insufficiency was indicated in COVID-19 patients through elevated blood dp-ucMGP levels [27], which were also associated with increased mortality risk in sex- and age-adjusted analyses [28]. Given that the carboxylation status of OC is a likely determinant of its functionality, there is a strong rationale for investigating specifically carboxylated OC during systemic inflammation as well as in response to exogenously boosted vitamin K levels. In a study on obese subjects, significantly lower circulating Gla-OC was detected as compared to non-obese individuals, indicating that Gla-OC levels inversely correlate to the development of inflammation during obesity [29]. This, together with our findings of increased Gla-OC after vitamin K1 administration in critically ill patients, supports the notion of vitamin K having counter-inflammatory effects through increasing carboxylation of extrahepatic VKDPs. In support of this possibility, a recent vitamin K2 supplementation trial on hospitalised COVID-19 patients showed that menaquinone-7 (MK-7) significantly decreased circulating levels of dp-ucMGP [30].

Injected vitamin K1 appears not to have influenced the carboxylation of prothrombin, as observed through no difference in the PIVKA-II assay. This concurs with our previous observation of no increase in thrombin generation assay after vitamin K1 treatment [19]. However, it should be noted that the concentration values for plasma PIVKA-II measured in our assay were relatively high, at least tenfold higher than Gas6 and OC. We previously observed that patients have increased PIVKA-II levels already at admission into ICU; moreover, their PIVKA-II levels increase further during ICU stay [12]. Therefore, subtle differences in carboxylation influenced by exogenous vitamin K may not have been captured in this assay.

The differences in the carboxylation status of different VKDPs in response to exogenous vitamin K administration can be due to several factors. One is the tissue site of origin of the particular VKDP, which would require the injected vitamin K to reach the cells producing the protein in sufficient amounts to stimulate cellular GGCX activity. Such stimulation would presumably only occur if there were an actual vitamin K deficit within the specific tissue. Indeed, such potential disparities might exist with what is deemed to be sufficient levels of vitamin K in the liver versus extrahepatic tissues, and is a topic that requires further investigation. In the normal adult population, vitamin K deficiency in the liver is not expected, thus all VKDP coagulation factors are expected to be fully carboxylated. However, this is in stark contrast to some extrahepatic VKDPs. For example, in non-K supplemented adults, OC and MGP are at 20% and 30% non-carboxylated levels, respectively [31,32]. Also, Gas6 has 11 Gla residues, many more than the other VKDPs (OC has three) and hence could be even more sensitive to partial carboxylation. Furthermore, while in extrahepatic tissues the VKOR oxidoreductase is inhibited by coumarins such as warfarin, the liver also possesses a coumarin-insensitive quinone reductase activity. This means that, whilst in the liver the haemostatic functions can be maintained at a certain ratio of warfarin to vitamin K1, in extrahepatic tissues warfarin might cause a functional vitamin K deficiency. Also, a general tendency towards under-carboxylation has been linked to ageing and decreasing vitamin K consumption in the Western world in recent decades [33]. These observations indicate that, whilst vitamin K1 in the body may be at RDI levels based on sufficiency for blood clotting, extrahepatic VKDPs may yet be under-carboxylated and hence not fully functional. This, therefore, provides a rationale for the quantification of modification of extrahepatic VKDPs through carboxylation in certain pathologies, including systemic inflammation in which several VKDPs may be dysregulated.

Another factor that may account for the differential response to injected vitamin K amongst the three VKDPs studied here is the form of vitamin K. In this study, we administered a synthetic form of vitamin K1. However, extrahepatic tissues are believed to favour vitamin K2 (menaquinones) [1]. Normally, after intestinal uptake, all K vitamins are transported to the liver within lipoproteins. Whilst most vitamin K1 is retained in the liver, K2 travels further to extrahepatic tissues. This difference could account for a particular effect of K1 vs. K2 on the carboxylation state of extrahepatic VKDPs. However, it was also shown that menaquinone-4 can be synthesised from phylloquinone through the action of the enzyme UBIAD1 [34]. Therefore, our observations indicate that injected vitamin K1 may have been converted to sufficient K2 to induce full carboxylation of OC but not of Gas6, which has a much broader expression profile. Nevertheless, a similar investigation into the effects of exogenously administered vitamin K2 would clarify the relative carboxylating potentials of both K vitamers. Currently, dietary reference values (DRVs) for vitamin K (all forms) could not be established based on available data and, therefore, adequate intakes (AIs) were set but for phylloquinone only (1 μg/kg body weight per day) whereas current evidence on menaquinones is insufficient [35]. In terms of endogenously synthesized vitamin K2, which occurs through gut bacteria mainly in the terminal ileum and colon [36], this process would be expected to be disrupted in ICU patients due to their impaired physiology and altered gut microbiome [37].

As regards the value of measuring blood vitamin K levels alongside VKDP measurements in such studies, blood vitamin K levels may not be a strong correlate of plasma VKDP levels. In our study, the iv-injected K1 is likely to have multiple metabolic fates and sites of action, including conversion to K2 and uneven distribution across different tissues. Also, injected vitamin K1 has a half-life of 1.7 hr and fell below detection limits after 10 h, whilst at the same time showing a long duration of action (>168 h) in terms of clotting factor synthesis [38]. This supports the notion that plasma K presence is transient and leads to more durable outcomes through the carboxylation of a variety of VKDPs across different organs.

There was a significant positive correlation between PIVKA-II and undercaboxylated OC (ucOC) levels. Such a relationship matches what is expected for two biomarkers of low vitamin K levels, even though both proteins are produced in different organs, prothrombin in the liver and OC in bone. A significant correlation between these two markers was first reported in healthy subjects [39] as well as subsequently in patients with cholestatic liver disease [40]. The present study presents evidence for such a relationship also in critically ill patients, which exists despite there being no changes in the plasma levels of either protein after K1 administration. The lack of correlations observed between all other markers may be due to even greater differences between those proteins, or the particular sensitivities of the various assays employed, for example where PIVKA-II levels correlated with ucOC but not Gla-OC. Therefore, it is plausible that VKDP undercarboxylation, as measured here through both PIVKA-II and ucOC, was captured with greater sensitivity than increased VKDP carboxylation (Gla-OC). Furthermore, as regards Gas6, in this study only total Gas6 was analysed for correlations as the Gla-Gas6 assay was performed on samples with equalised total Gas6 levels. Moreover, correlation analysis for Gas6 total levels and a specifically carboxylated VKDP might be less relevant than for the other correlations as the mechanism by which vitamin K1 affects Gas6 total levels is likely different from its GGCX carboxylase cofactor role.

We recognise the limitations in the present study given its ‘before and after’ design. This is partly addressed by additional analyses that describe a possible direct effect of the vitamin K1 administration, but a time-dependent bias cannot be ruled out. Furthermore, the study is explorative only, aiming to describe changes in plasma PIVKA-II, ucOC, total Gas6 and carboxylated Gas6 levels 20–28 h after vitamin K1 administration and does not describe any positive or negative clinical effects of these changes. Therefore, repetitive analyses over longer intensive care or postoperative periods and repeated doses of vitamin K1 and ELISAs for detection of effects on carboxylation are needed to take this research further. We previously performed studies on repetitive plasma analyses on PIVKA in the postoperative period, showing an increased trend in subclinical vitamin K deficiency [41]. Furthermore, the potential risk of increasing Gas6 carboxylation with vitamin K and its effects on thromboembolism and cancer cell spread also needs to be considered [16].

## 5. Conclusions

This study has shown that injected vitamin K1 increases the circulating levels of two distinct VKDPs in critically ill patients, including carboxylated OC. As several VKDPs have been implicated in inflammation, these results suggest that restoring vitamin K imbalance in such patients may play a role in their clinical outcomes. However, in order to determine the beneficial role of boosting vitamin K in such patients, it will be necessary to conduct further investigations including randomised controlled studies and analysing biomarkers of inflammation alongside clinical outcomes. 

## Data Availability

The original contributions presented in the study are included in the article, further inquiries can be directed to the corresponding author.

## Supplementary Materials

The following supporting information can be downloaded at: https://www.mdpi.com/article/10.3390/nu17020348/s1, Table S1. Assay results on patient subgroups according to disease category.
