# Supplementary material for: Vitamin K1 Administration Increases the Level of Circulating Carboxylated Osteocalcin in Critically Ill Patients
Source: Nutrients. 2025 Jan 19;17(2):348. doi: 10.3390/nu17020348 (PMC11768845; doi:10.3390/nu17020348)
Supplement: Supplementary file 1 [file nutrients-17-00348-s001.zip › nutrients-3418586-supplementary.pdf]

Aydin *et al*. 2025 - Supplementary Table S1

Assay results on patient subgroups according to disease category

	**Septic shock (n = 14)**	**Cardiovascular disease (n = 6)**	**Cancer (n = 2)**
**Total Gas6** (Paired t- test)	p = 0.1993	p = 0.8689	p = 0.8202
**Carboxylated Gas6 (Gla-Gas6)** (Wilcoxon)	p = 0.5416	p = 0.3125	
**Gla-OC** (Wilcoxon)	*p = 0.0353	p = 0.2188	
**ucOC** (Paired t- test)	p = 0.8563	p = 0.3078	p = 0.0936
**PIVKA-II** (Paired t- test)	p = 0.8377	p = 0.7685	p = 0.1326
